# Impact of CD11c^+^ cells in conducting airway lumen on *Aspergillus fumigatus* conidia deposition in neutropenic mice

**DOI:** 10.3389/ffunb.2025.1591891

**Published:** 2025-06-18

**Authors:** Mariia Pavelchenko, Svyatoslav Shalyapin, Sergey Portnov, Andrey Bogorodskiy, Elena Bolkhovitina, Vitalii Shevchenko, Alexander Sapozhnikov, Valentin Borshchevskiy, Marina Shevchenko

**Affiliations:** ^1^ Laboratory of Cell Interaction, Immunology Department, Shemyakin and Ovchinnikov Institute of Bioorganic Chemistry, Russian Academy of Sciences, Moscow, Russia; ^2^ Research Center for Molecular Mechanisms of Aging and Age-Related Diseases, Moscow Institute of Physics and Technology, Dolgoprudny, Russia; ^3^ Department of Translational Medicine, National Research Nuclear University MEPhI (Moscow Engineering Physics Institute), Moscow, Russia; ^4^ Department of Applied Mathematics, Tikhonov Moscow Institute of Electronics and Mathematics National Research University Higher School of Economics, Moscow, Russia; ^5^ Frank Laboratory of Neutron Physics, Joint Institute for Nuclear Research, Dubna, Russia

**Keywords:** neutrophil depletion, invasive aspergillosis mouse model, confocal laser-scanning microscopy, whole-mount lung imaging, *Aspergillus fumigatus* conidia distribution, conducting airway mucosa, immune cell–pathogen interactions, macrophages

## Abstract

**Introduction:**

Inhaled conidia of the opportunistic fungi *Aspergillus fumigatus* settle in the airway mucosa and in alveolar spaces. Different immune cells typically provide crucial defense against fungal germination. However, in immunocompromised patients, the lack of sufficient pro-inflammatory immune response often leads to invasive aspergillosis, with current treatments being limited by insufficient understanding of the precise conidial distribution patterns in the airways.

**Methods:**

Therefore, we employed advanced imaging techniques, including immunohistochemistry, optical clearing, and confocal laser scanning microscopy, to map *A. fumigatus* conidial distribution in both immunocompetent and neutropenic mouse airways. We developed a 3D airway model distinguishing the main bronchus, intermediate bronchi, and terminal bronchioles, enabling quantitative analysis of conidial location. In addition, we analyzed the interactions of CD11c^+^ cells with conidia in the conducting airway mucosa.

**Results:**

Our findings revealed that while the majority of conidia reached the alveolar space in both groups, neutropenic mice showed significantly higher conidial concentrations in bronchial branches, particularly in the main bronchus, compared with immunocompetent mice. Simultaneously, in the conducting airway mucosa of neutropenic mice, CD11c^+^ cells ingested an elevated number of conidia compared with immunocompetent mice.

**Discussion:**

Thus, detailed mapping of the conidial distribution patterns provides crucial insights into the spatial aspects of antifungal treatment in neutropenic patients. The enhanced contribution of CD11c^+^ cells to conidial internalization in the conducting airway mucosa of neutropenic mice demonstrated in the present study emphasizes the potential of these cells in the development of more effective, cell-targeted antifungal treatments.

## 1 Introduction

Conidia of the fungus *Aspergillus fumigatus* are common in both indoor and outdoor environments (O’Gorman, 2011). In environmental conditions, conidia exist in a dormant or resting state and are therefore initially inert to the human immune system when inhaled. They can reside in hidden niches until germination (Aimanianda et al., 2009). Whether the locations of conidia are the same in both immunocompetent and immunocompromised organisms remains unclear. However, upon germination, *A. fumigatus* conidia are eliminated in immunocompetent individuals. In contrast, in patients with congenital neutropenia, a disorder characterized by impaired neutrophil function, or in those with acquired neutropenia due to immunosuppressive therapy, conidia can induce invasive aspergillosis. This condition results in a high mortality rate despite the availability of diagnostics and antifungal medications (Brown et al., 2012; Gazendam et al., 2016; Van De Veerdonk et al., 2017; Wang et al., 2022; Janssens et al., 2024).

The antifungal immune response, known as type 3 immunity, is mediated by ILC3 (group 3 innate lymphoid cells) and Th17 (T helper 17). These cells secrete interleukin 17 (IL-17) and IL-22 to stimulate neutrophil recruitment (Annunziato et al., 2015). In immunocompetent mice, neutrophils infiltrate the airways as soon as 6 h after oropharyngeal (o.ph.) administration of *A. fumigatus* conidia, supporting the possibility of direct neutrophil activation by sensor cells (Iwasaki et al., 2017; Shevchenko et al., 2018; Bogorodskiy et al., 2020). Upon inhalation, *A. fumigatus* conidia distribute across the airways from the trachea to the alveolar compartment, with different immune sensor cell subsets recognizing conidia in different airway generations (Amich et al., 2020; Maslov et al., 2021). Experiments using mouse models have demonstrated that immune sensor cells are predominantly represented by macrophages and dendritic cells located on the luminal side of the airway epithelial barrier (Amich et al., 2020; Bogorodskiy et al., 2020). Both of these populations express receptors for recognizing growing conidia: C-type lectin receptors (e.g., Dectin-1/2, Clec4d, Clec4e, and mannose receptor), along with Toll-like receptors (e.g., TLR2 and TLR4) (Li et al., 2019b). Simultaneously, these cells can internalize inert conidia as they express phagocytosis receptors, the complement receptor 3 (CR3) (CD11b/CD18) and CR4 (CD11c/CD18) (Lukácsi et al., 2017).

The alpha chain of CR4—the transmembrane glycoprotein CD11c—also serves as a marker for macrophages and dendritic cells. It is used in combination with other markers for the detection of macrophage and dendritic cell subsets via flow cytometry, as well as for visualization in tissue sections and organs (Zoltán Veres et al., 2011; Guilliams et al., 2016; Bošnjak et al., 2022). CD11c-enhanced yellow fluorescent protein (EYFP) mice were employed to investigate the role of intraepithelial dendritic cells in the immune response induced by *A. fumigatus* conidia (Bogorodskiy et al., 2020). At the same time, CD11c.DTR mice were used to examine the role of alveolar macrophages during *A. fumigatus* infection (Männ et al., 2016). Even though macrophages and dendritic cells cannot provide complete protection from *A. fumigatus* infection without neutrophils, they can inhibit conidial germination, suggesting their potential as target cells for the development of antifungal therapy (Mircescu et al., 2009; Lother et al., 2014; Rosowski et al., 2018; Idol et al., 2022). As different subsets of macrophages and dendritic cells reside across the airways, a precise analysis of the conidial distribution and the interactions between immune cells and conidia is needed to identify the location and characterize the advanced populations.

In this study, we aimed to compare the spatial distribution of *A. fumigatus* conidia in the airways of both immunocompetent mice and mice with neutrophil depletion. To do this, immunohistochemical staining and optical clearing of whole-mount lung lobe specimens were carried out. We elaborated an approach for image processing that made it possible to analyze the location of the fluorescently labeled *A. fumigatus* conidia relative to the different airway generations: the main bronchus, the intermediate bronchi, the terminal bronchioles, and the alveolar space. A noticeable difference in the proportions of conidia deposited in the bronchi (particularly at 48 h after administration in the main bronchus) between immunocompetent mice and mice with neutrophil depletion was observed. Using CD11c-EYFP mice, we also assessed the impact of ingestion of *A. fumigatus* conidia by the conducting airway CD11c^+^ cells on the conidial deposition in the main bronchus of both immunocompetent mice and mice with neutrophil depletion.

## 2 Materials and methods

### 2.1 Animals and ethics statement

Male CD11c-EYFP mice (18–30 weeks old) on C57BL/6 background (Lindquist et al., 2004), kindly gifted by Prof. Armin Braun (Fraunhofer Institute for Toxicology and Experimental Medicine ITEM, Hannover, Germany) and bred in the animal facility of Shemyakin and Ovchinnikov Institute of Bioorganic Chemistry, Russian Academy of Sciences, were used in this study. All animal experiments were performed in concordance with the Guide for the Care and Use of Laboratory Animals under a protocol approved by the Institutional Animal Care and Use Committee at Shemyakin and Ovchinnikov Institute of Bioorganic Chemistry, Russian Academy of Sciences (protocol nos. 245/2018 and 383/2024). Animals were given standard food and tap water *ad libitum* and housed under regular 12-h dark/light cycles at 22°C.

### 2.2 Neutrophil depletion

Mice received an intraperitoneal (i.p.) injection of rat anti-mouse Gr-1 antibodies (BioLegend, San Diego, CA, USA), 100 μg per mouse. The antibody dosage was chosen based on our previous findings (Shevchenko et al., 2018). The control groups received rat IgG2b (cat. no. 400622, RRID: AB_326564; BioLegend), 100 μg per mouse. All antibodies and isotype controls were diluted in Dulbecco’s phosphate-buffered saline (DPBS) (PanEco, Moscow, Russia) to a total volume of 200 μl and injected in mice 1 day before the o.ph. administration of *A. fumigatus* conidia.

### 2.3 *A. fumigatus* labeling and application

*A. fumigatus* conidia, strain AfS150, were kindly gifted by Prof. Sven Krappmann (University Hospital Erlangen and FAU Erlangen-Nürnberg, Germany). Conidia were fixed with paraformaldehyde (PanReac, Barcelona, Spain) as described previously (Maslov et al., 2021) and dissolved in DPBS to a concentration of 1 × 10^8^ conidia/ml. Mice were anesthetized by inhalation of isoflurane (Baxter, Guayama, Puerto Rico), and a 50-μl droplet containing 5 × 10^6^ conidia was administered to the oropharyngeal cavity of each mouse (Rao et al., 2003).

### 2.4 Blood collection and cell analysis

The peripheral blood was collected from the tail vein into 1.5-ml test tubes with 50 µl heparin (VelPharm, Kurgan, Russia) and transferred into 10 ml of the hemolysis buffer [155 mM NH_4_Cl (Reachem, Moscow, Russia), 0.1 mM Na_2_EDTA (Sigma-Aldrich, Steinheim, Germany), and 10 mM NaHCO_3_ (PanReac, Barcelona, Applichem), pH 7.3], stored at +4°C, and warmed to room temperature (RT) before use in 50-ml tubes. The samples were held for 5 min at RT, and then 20 ml of DPBS was added and centrifuged at 380 × *g* for 5 min. The supernatants were replaced with 5 ml of the hemolysis buffer. The samples were held for 5 min at RT, and then 20 ml of DPBS was added and centrifuged at 380 × *g* for 5 min. The pellet was transferred into 500 µl of the cytometry buffer [1% bovine serum albumin (BSA; Serva, Heidelberg, Germany) and 2 mM EDTA]. Subsequently, the cells were centrifuged using CV-1500 (Biosan, Riga, Latvia) for 10 min. The cell pellet was dissolved in 30 µl of the cytometry buffer and transferred into a 96-well plate.

For the estimation of the total blood leukocyte numbers, heparinized blood was diluted 1:3 with 3% acetic acid (Merck, Darmstadt, Germany). The cell nuclei were quantified within 30 min using the Goryaev chamber (Minimed, Bryansk, Russia).

For the neutrophil detection by flow cytometry, the method recommended by Liu et al. (2020) was utilized. The samples were preincubated with anti-mouse CD16/CD32 (cat. no. 130-092-574, RRID: AB_871624; Miltenyi Biotec, Bergisch Gladbach, Germany) for 15 min. Afterward, the following antibodies (all from Miltenyi Biotec) were used: anti-mouse Ly6G–VioBlue (cat. no. 130-119-902, RRID: AB_2751917), anti-mouse FcϵR1–PE (cat. no. 130-118-896, RRID: AB_2801720), anti-mouse SiglecF–PE-Vio615 (cat. no. 130-112-330, RRID: AB_2653443), anti-mouse CD172–PE-Vio770 (cat. no. 130-123-154, RRID: AB_2802004), anti-mouse CD45–APC-Vio770 (cat. no. 130-110-800, RRID: AB_2658230), and anti-mouse CD11b–VioGreen (cat. no. 130-113-811, RRID: AB_2726328). The antibodies were used at 1:30 dilution. The samples were incubated for 30 min and washed twice with DPBS. SytoxGreen (cat. no. S34859; Invitrogen, Carlsbad, CA, USA) was added (at 1:1,000,000 dilution) 5 min before the acquisition. Measurements were performed using a MACSQuant Analyzer 10 (RRID: SCR_020268; Miltenyi Biotec). For the quantitative analysis, 10,000 events were collected in the gate of leukocytes.

### 2.5 Lung harvesting

Animals were euthanized, and their lungs were harvested and fixed without inflation with 2% paraformaldehyde overnight at +4°C. The lungs were separated into lobes: the left and right inferior lobes were used for whole-mount conducting airway preparation and immunohistochemistry. The right superior lobes were stained as whole mounts for the analysis of conidial distribution.

### 2.6 Whole-mount lung lobe specimen preparation, staining, and optical clearing

The lung lobes were rinsed five times with Tris-buffered saline (TBS), pH 7.4, each rinse lasting for 1 h. Subsequently, the samples were blocked overnight with 0.3% Triton X-100 (Helicon, Moscow, Russia) and 5% powdered milk (Roth, Karlsruhe, Germany) in TBS at RT at 150 rpm on a shaker (Apexlab, Moscow, Russia). The airways were tagged with streptavidin conjugated to Alexa Fluor 488 (cat. no. S111223; Thermo Fisher, Waltham, MA, USA) for 3 days. The specimens were then rinsed in TBS and underwent overnight post-fixation in 2% paraformaldehyde (Scott et al., 2014). Optical clearing of the lung lobes was performed at RT on a sample mixer MXIC1 (Thermo Fisher) using a Ce3D Tissue Clearing Kit (cat. no. 427702, BioLegend), in line with both the manufacturer’s instructions and earlier studies (Li et al., 2019a). The lung lobes were then placed into cell imaging coverglass chambers (Eppendorf, Hamburg, Germany) and preserved until microscopy examination.

### 2.7 Whole-mount conducting airway specimen preparation and staining

The main bronchi from the lung lobes (left and right inferior) were dissected. Thereafter, the airways were washed with DPBS, permeabilized with 0.3% Triton X-100, and blocked with 1% BSA. Allophycocyanin (APC)-conjugated anti-mouse CD11b (cat. no. 101212, RRID: AB_312795; BioLegend) was used at 1:50 dilution. Phalloidin-Atto 425 (cat. no. 66939; Sigma-Aldrich, St. Louis, MO, USA) was used at 1:30 dilution. All samples were mounted in Prolong Gold mounting medium (cat. no. P36930; Thermo Fisher).

### 2.8 Confocal laser scanning microscopy of whole-mount lung lobe

Imaging of mouse lung lobe was conducted as previously described by us (Maslov et al., 2021). An inverted LSM780 confocal microscope (Zeiss, Jena, Germany) with a ×10 (NA = 0.3) objective was used. Excitation at 488 and 594 nm was employed to image the fluorescence of Alexa Fluor 488 and Alexa Fluor 594, respectively. The emission was gauged in confocal laser scanning microscopy (CLSM) *λ*-mode utilizing a 34-channel QUASAR detector (Zeiss), which was set to a range of 490–695 nm. The ZEN 2012 SP5 software (Zeiss) was used to perform spectral unmixing. Images were taken in Tile-Scan mode as *Z*-stacks, with a resolution of 512 × 512 and a *Z*-step of 5 µm.

### 2.9 CLSM of whole-mount conducting airways

For the visualization of whole-mount conducting airways, an inverted confocal LSM780 microscope (Zeiss) equipped with a ×40 (NA = 1.4, water immersion) objective was employed. The quantitative analysis of CD11c cell–*A. fumigatus* conidia interaction utilized excitation at 405, 488, and 594 nm for observing Atto 425, EYFP, and Alexa Fluor 594, respectively. The emission was gauged in CLSM *λ*-mode, with a 34-channel QUASAR detector set for the 405- to 695-nm range. Spectral unmixing was accomplished with the help of ZEN 2012 SP5 software. Images were acquired in the form of *Z*-stacks with dimensions of 354.25 µm × 354.25 µm × 40 µm.

### 2.10 3D Model airway quantitative image analysis

An airway surface was created based on the maximum intensity projection using Imaris version 9.8 software. This surface was then corrected using FIJI software (Schindelin et al., 2012) and transferred back to Imaris, a process we have described in detail previously (Maslov et al., 2021). The surface was divided into structural elements of roughly 20 µm in size. These elements were manually classified as the main bronchus, the intermediate bronchi, or the terminal bronchioles. Mask channels were created for the corresponding surfaces. *A. fumigatus* conidia were processed as 5-µm-sized spots, with point spread function (PSF) elongation along the *z*-axis measuring 10 µm. Using the “Quality” (intensity at the center of the spot) filter in the corresponding (conidia) channel, the conidia were classified as either inside or outside the bronchial tree. From those categorized as inside, conidia were further distinguished as being in the main bronchus, the intermediate bronchi, or the terminal bronchioles.

### 2.11 Quantitative analysis of conducting airway images

Image stacks were analyzed using Imaris software. CD11c cells were identified and processed with a “three-dimensional surface rendering” of the appropriate channel based on the maximum intensity projection, as previously described (Veres et al., 2007). The thresholds and filter settings were optimized through visual control. The estimated diameter of the CD11c cell was set to 20 µm. The numbers of cells were automatically calculated from their respective surface objects. The location of cells was determined in relation to the epithelial and smooth muscle barriers, visualized according to the actin filament phalloidin staining. *A. fumigatus* conidia were processed as spots with a diameter of 3 µm and a *z*-axis PSF of 6 µm. The number of conidia within the CD11c cell was estimated using the “Intensity Mean” filter in the EYFP channel.

### 2.12 Statistical analysis

The data are presented as the median and interquartile range (IQR). For small datasets (*n* = 4 mice) and two-group comparisons, the Mann–Whitney test was employed for statistical analysis. For the comparisons of three or more groups, one-way analysis of variance (ANOVA) (Kruskal–Wallis) and Dunn’s multiple comparison tests were utilized. Datasets with *n* ≥ 8 mice underwent initial analysis for normal data distribution using the Shapiro–Wilk test. For datasets with a normal distribution and three or more groups, one-way ANOVA and Dunnett’s multiple comparison tests were applied. Analysis was performed using GraphPad Prism 7 software (GraphPad Software, San Diego, CA, USA). A *p*-value less than 0.05 was considered to be statistically significant.

## 3 Results

### 3.1 Airway branch generation modeling

**Figure 1:**
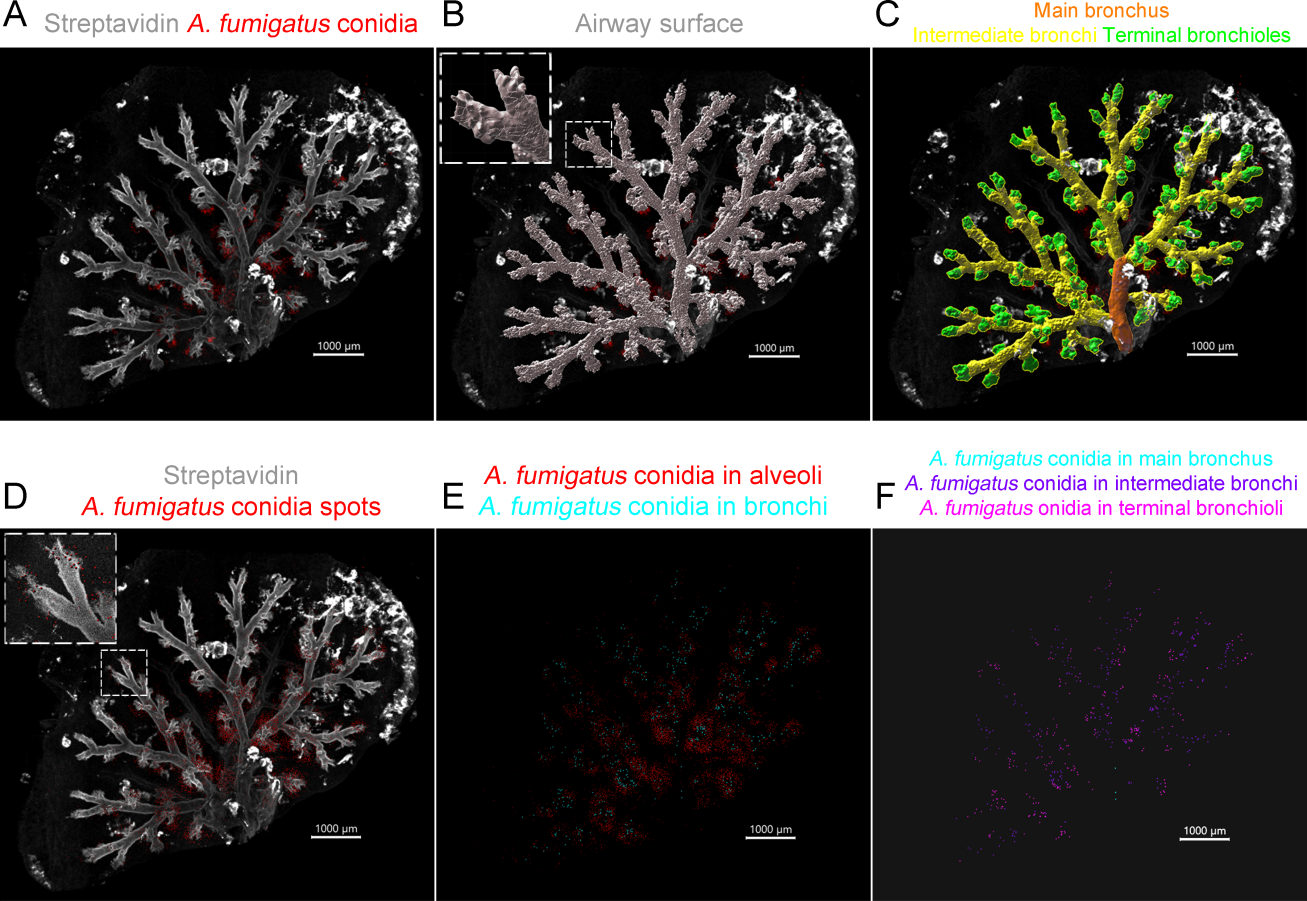
Classification of the airway generations and *Aspergillus fumigatus* conidia in the respective airway generations. **(A)** Representative image of the optically cleared mouse lung lobe 48 h after the oropharyngeal (o.ph.) administration of *A. fumigatus* conidia. Streptavidin (*grayscale*) and *A*. *fumigatus* conidia (*red*) are represented via volume rendering. **(B)** The surface of the airways was built and split into elements with an approximate diameter of 20 µm (for details, see the enlarged fragment in the *insertion*). **(C)** The elements were grouped into the main bronchus (*orange*), the intermediate bronchi (*yellow*), and the terminal bronchioles (*green*). **(D)** *A*. *fumigatus* conidia are presented as *spots* (for details, see the enlarged fragment in the *insertion*). **(E)** Conidia were classified as inside the bronchial branches (*cyan*) and outside, in the alveolar space (*red*). **(F)** Conidia were classified as in the main bronchus (*cyan*), in the intermediate bronchi (*violet*), and in terminal bronchioles (*magenta*). *Spots* are presented with a radius scale of 5 µm **(D–F)**. *Scale bar*, 1,000 µm.

The following experimental setup was utilized to examine the distribution of *A. fumigatus* conidia in the airways of mice. *A. fumigatus* conidia were labeled with Alexa Fluor 594 before their o.ph. administration to mice. After 6 and 48 h of the extraction of whole-mount lung lobes, they were stained with Alexa Fluor 488-conjugated streptavidin and underwent optical clearing. Images were captured using CLSM in *Z*-stack tiles and later processed with Imaris software to construct a 3D model of the airway tree (Maslov et al., 2021). For the classification of the airway branches into generations, the surface of the airway tree was divided into elements measuring 20 µm (**Figures 1A, B**; **Supplementary Figure S1**). These elements were manually grouped into the following categories: main bronchus, intermediate bronchi, and terminal bronchioles (**Figure 1C**).

To quantify *A. fumigatus* conidia in the optically cleared mouse lung lobes, conidia were depicted as spots (**Figure 1D**). Initially, we differentiated between conidia located inside and outside the bronchial branches (**Figure 1E**). Those found outside were classified as being located in the alveolar space, as previously reported (Maslov et al., 2021). Subsequently, we created masks for the airway generations based on their surfaces (**Supplementary Figure S1**). Conidia that were identified inside bronchial branches were categorized using the “Intensity Mean” filter in the corresponding mask channel. Based on this classification, conidia were ranked as being located in the main bronchus, the intermediate bronchi, and the terminal bronchioles (**Figure 1F**).

Thus, dividing the airway surfaces into elements allowed accurately classifying the airway generations into the main bronchus, the intermediate bronchi, and the terminal bronchioles and then determining the conidia location in relation to these generations.

### 3.2 Anti-Gr-1 antibody induces prolonged depletion of blood myeloid cells and neutrophils

**Figure 2:**
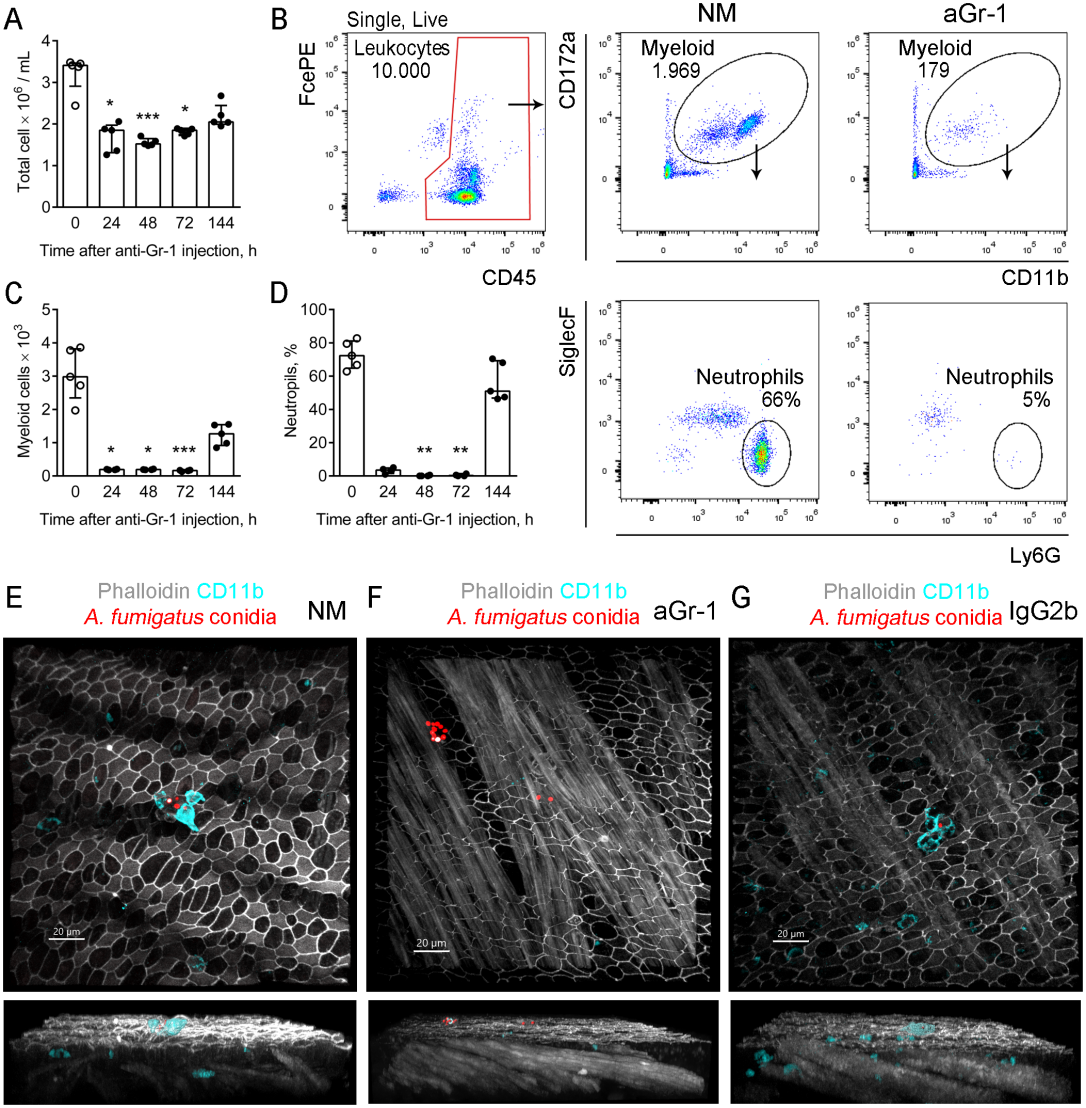
Kinetics of neutrophil depletion using anti-Gr-1 antibodies. **(A)** Total blood cell number detected by nuclei count after treatment with acetic acid at different time points after the depleting antibody injection. Data are shown as median and IQR, *n* ≥ 4 mice per group. Significant differences between the indicated time point and time point 0 (untreated mice) were detected using the Kruskal–Wallis and Dunn’s multiple comparison tests. *p ≤ 0.05, ***p ≤ 0.005. **(B)** Representative flow cytometry plots showing the strategy for identifying the neutrophil population in the peripheral blood of untreated mice (*NM*) and mice that received depleting antibodies (*aGr-1*). **(C)** Myeloid cell counts were obtained using the strategy presented in **(B)** at different time points after injection of the depleting antibody. Data are shown as median and IQR (*n* ≥ 4 mice per group). Significant differences between the indicated time point and time point 0 (untreated mice) were detected using the Kruskal–Wallis and Dunn’s multiple comparison tests. *p ≤ 0.05, ***p ≤ 0.005. **(D)** Proportions of neutrophils among myeloid cells at the different time points after injection of the depleting antibody. Data are shown as median and IQR (*n* ≥ 4 mice per group). Significant differences between the indicated time point and time point 0 (untreated mice) were detected using the Kruskal–Wallis and Dunn’s multiple comparison tests. **p ≤ 0.01. **(E–G)** Representative images of the conducting airway mucosa of immunocompetent mice **(E)**, mice that received depleting antibodies **(F)**, and control mice **(G)** 48 h after the oropharyngeal (o.ph.) administration of *Aspergillus fumigatus* conidia and 72 h after treatment with antibodies. Neutrophil (*cyan*) and conidia (*red*; radius scale, 3 μm) are shown via volume rendering in the frontal views (*upper images*) and as surfaces in the side views (*lower images*). Actin (*grayscale*) is shown via volume rendering. *Scale bar*, 20 μm.

To compare the distribution of *A. fumigatus* conidia in immunocompetent and immunocompromised mice, one group of mice was subjected to the induction of neutropenia. Neutropenia was simulated by injecting the mice with rat anti-mouse Gr-1 antibodies. Subsequent total blood leukocyte quantification via the traditional method using acetic acid showed a notable reduction in cell numbers at 24, 48, and 72 h post-injection (**Figure 2A**). However, on day 6 after injection, the cell numbers did not significantly differ from those of untreated mice, indicating a steady leukocyte repopulation.

To more precisely assess the effect of the injection on blood myeloid cells and neutrophils, the following gating strategy was implemented (**Figure 2B**; **Supplementary Figure S2**). For comparison, the number of leukocytes was set to 10,000 events (**Figure 2B**, the gate is indicated in red). The kinetics of both blood myeloid cell numbers and neutrophil percentages demonstrated similarities to that of total blood leukocytes. The number of myeloid cells and the neutrophil percentages significantly decreased up to 72 h following injection of the depleting antibody, but an increase was observed on day 6 (**Figures 2C, D**).

Neutrophil recruitment was also examined in the conducting airway mucosa of immunocompetent, neutropenic, and control mice 48 h after the o.ph. administration of *A. fumigatus* conidia and 72 h after treatment with antibodies (**Figures 2E–G**). Notably, at this time point, the peripheral blood neutrophil count in anti-Gr-1-treated mice was still significantly reduced compared with that in immunocompetent mice (**Figure 2D**). In a previous study, we demonstrated that the neutrophil numbers in the conducting airway mucosa of immunocompetent mice were diminished 48 h after the o.ph. administration of conidia compared with the counts at 12 and 24 h (Bogorodskiy et al., 2020). Nevertheless, in immunocompetent and control mice, the neutrophils interacted with conidia, whereas no neutrophils were detected in the conducting airway mucosa of neutropenic mice (**Figures 2E–G**). To prevent signal loss due to the blocking of the Ly6G receptor by the anti-Gr-1 depleting antibodies, neutrophil visualization was performed using anti-CD11b antibodies.

Intraperitoneal administration of anti-Gr-1 depleted the myeloid cells and neutrophils from the peripheral blood and conducting airway mucosa for a prolonged period of at least 72 h.

### 3.3 *A. fumigatus* conidia sediment in bronchial brunches of neutropenic mice

To estimate the distribution of *A. fumigatus* conidia in the airways of mice, the experimental setup outlined above was implemented. During conidia imaging and depiction, the intensity threshold value had a strong impact on the number of detected conidia: at low thresholds, the autofluorescence from lung tissue could be mistakenly identified as conidia. To minimize this error, the proportions of depicted conidia were compared across different threshold values. For the thresholds that correspond to the plateau region on the intensity histogram, a minimal influence of the threshold value on the conidia proportion was observed (**Supplementary Figure S3**).

**Figure 3:**
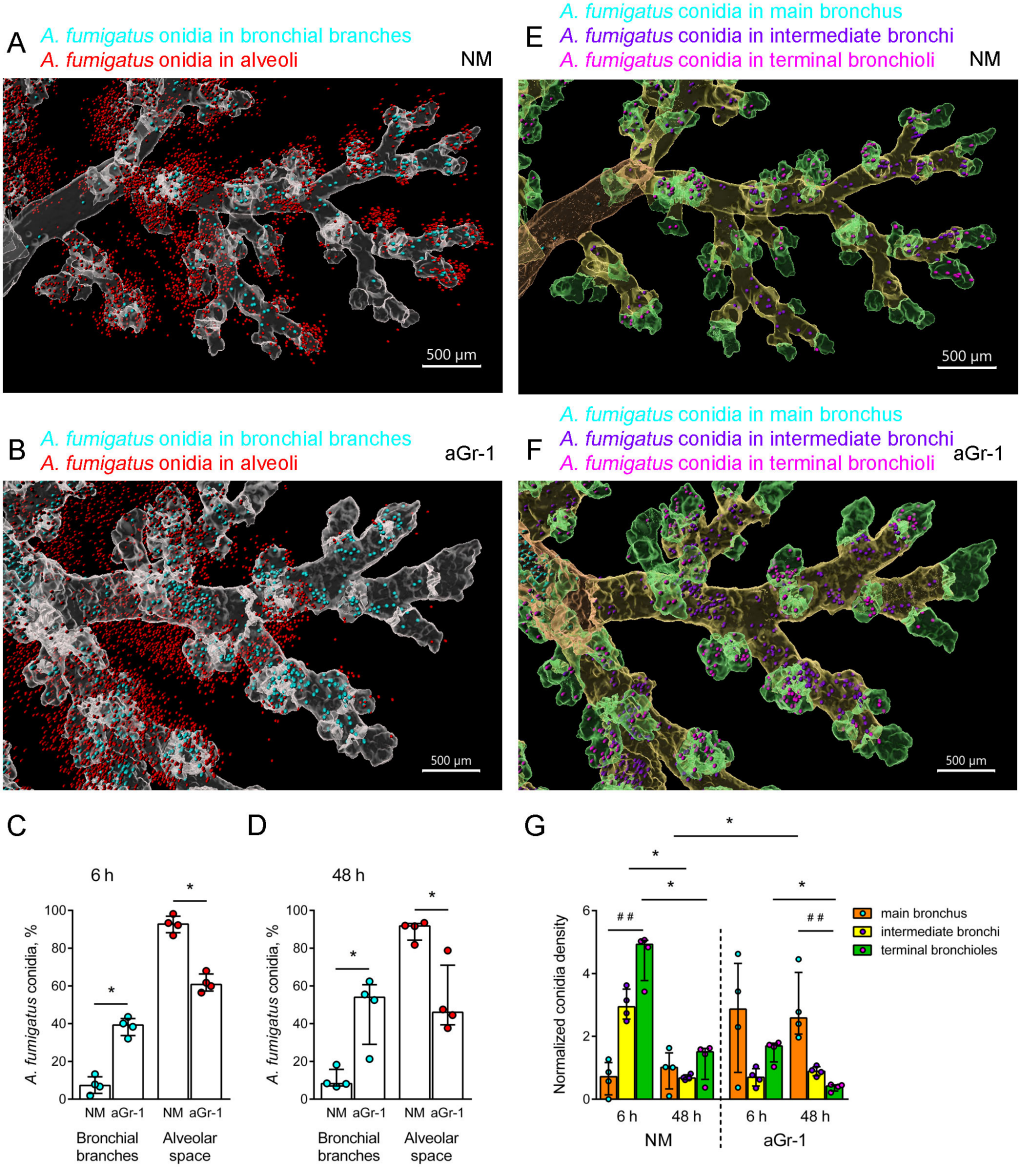
Distribution of *Aspergillus fumigatus* conidia in the airways of immunocompetent and neutropenic mice. **(A, B)** Representative images of the fragments of the conducting airways (*grayscale*, *transparent*) and conidia inside the bronchial branches (*cyan*) and outside, in the alveoli (*red*) of immunocompetent (*NM*) **(A)** and neutropenic (*aGr-1*) **(B)** mice 48 h after the oropharyngeal (o.ph.) administration of conidia. *Scale bar*, 500 μm. **(C, D)** Percentages of conidia in the bronchial branches (*cyan circles*) and the alveolar space (*red circles*) of NM and aGr-1 mice 6 h **(C)** and 48 h **(D)** after the o.ph. administration of conidia. Data are shown as median and IQR, *n* = 4 mice per group. Significant differences between groups were determined using the Mann–Whitney test. *p ≤ 0.05. **(E, F)** Representative images of the fragments of conducting airways split into generations: main bronchus (*orange*, *transparent*), intermediate bronchi (*yellow*, *transparent*), terminal bronchioles (*green*, *transparent*), and conidia inside the main bronchus (*cyan*), intermediate bronchi (*violet*), and terminal bronchioles (*magenta*) in immunocompetent (*NM*) **(E)** and neutropenic (*aGr-1*) **(F)** mice 48 h after the o.ph. administration of conidia. *Scale bar*, 500 μm. *Spots* inside the airway branches are presented with a radius scale of 7 µm and those outside of 5 µm **(A, B, E, F)**. **(G)** Normalized *A*. *fumigatus* conidia density in the main bronchus, intermediate bronchi, and terminal bronchioles of NM and aGr-1 mice 6 and 48 h after the o.ph. administration of conidia. Data are shown as median and IQR (*n* = 4 mice per group). Significant differences between generations within each group (NM or aGr-1) for each time (6 or 48 h) were determined using the Kruskal–Wallis and Dunn’s multiple comparison tests (^##^ *p* ≤ 0.01). Pairwise comparisons of the indicated groups were performed using the Mann–Whitney test (*p ≤ 0.05).

Initially, the percentages of *A. fumigatus* conidia in the bronchial branches and alveolar spaces of immunocompetent mice were estimated (**Figure 3A**; **Supplementary Figure S3**). To trace conidial distribution early after inhalation and upon elimination from the respiratory tract, an extended time range—6, 48, and 72 h after the o.ph. administration of conidia—was selected based on previous studies (Buskirk et al., 2014; Savers et al., 2016; Bogorodskiy et al., 2020). The percentages of conidia were higher in the alveolar spaces at all examined time intervals (**Supplementary Figure S3**). The distribution of *A. fumigatus* conidia in immunocompetent mice was then compared with that in neutropenic mice (**Figures 3A, B**). In neutropenic mice, the percentages of conidia in the bronchial branches significantly exceeded those in immunocompetent mice 6 and 48 h after the o.ph. administration of conidia (**Figures 3C, D**).

A detailed analysis of the distribution of *A. fumigatus* conidia was performed within 6–48 h after o.ph. administration, focusing on the main bronchus, the intermediate bronchi, and the terminal bronchioles of both immunocompetent and neutropenic mice (**Figures 3E, F**). Each airway generation has a different volume, and the volume of each generation varies across individual samples, which can also influence the number of deposited conidia (**Supplementary Figure S4**). To account for these factors and enable meaningful comparison of the conidial density across generations, we calculated the normalized *A. fumigatus* conidial density as follows:


$$\text{Normalized conidia density}=\frac{\frac{\text{Number of conidia in generation }x}{\text{Volume of generation }x}}{\frac{\text{Total number of conidia in bronchi}}{\text{Total bronchi volume}}}$$

Varying profiles of conidial distribution were observed between immunocompetent and neutropenic mice (**Figure 3G**). An early (6 h) increase in the distribution densities of *A. fumigatus* conidia in the intermediate bronchi and terminal bronchioles was noted, which significantly decreased 48 h after their application (**Figure 3G**), suggesting successful clearance of conidia from the respiratory tract of immunocompetent mice. The same kinetics was observed for the terminal bronchioles of neutropenic mice (**Figure 3G**). However, in the main bronchus of neutropenic mice, the proportion of conidia increased at 6 h and was significantly elevated at 48 h after their o.ph. administration compared with immunocompetent mice (**Figure 3G**). This indicates the retention of conidia in the main bronchus under conditions of neutropenia.

*A. fumigatus* conidia were primarily found in the alveolar spaces of immunocompetent mice, while they were predominantly located in the bronchial branches in mice with induced neutropenia.

### 3.4 *A. fumigatus* conidia are stuck in the conducting airway mucosa of neutropenic mice

**Figure 4:**
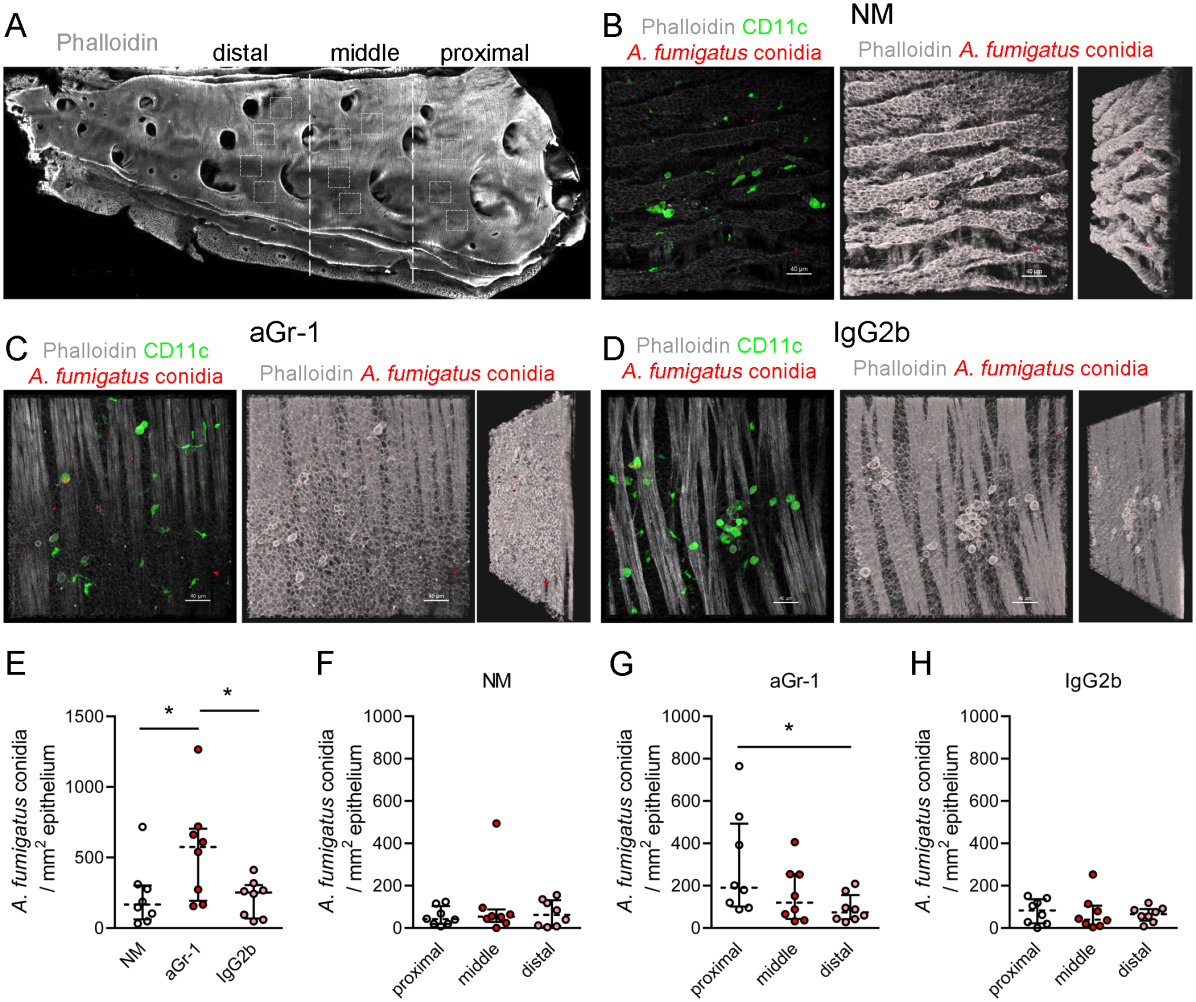
Distribution of *Aspergillus fumigatus* conidia in the conducting airways of immunocompetent and neutropenic mice 48 h after oropharyngeal (o.ph.) administration. **(A)** Representative image of whole-mount conducting airway of an immunocompetent mouse (actin fibers in *grayscale*). The proximal, middle, and distal airway segments are bordered with *dotted lines*. The regions of image acquisitions are arbitrarily indicated with *dotted squares*. *Scale bar*, 1,000 µm. **(B–D)** Representative images from the proximal regions of the conducting airways of immunocompetent (*NM*) **(B)**, neutropenic (*aGr-1*) **(C)**, and control (*IgG2b*) **(D)** mice 48 h after o.ph. administration of *A*. *fumigatus* conidia. *Left images* demonstrate CD11c^+^ cells (*green*) and conidia (*red*) in the conducting airway mucosa, bordered by the epithelium and smooth muscles, visualized by actin fibers (*grayscale*) via volume rendering. The *middle images* demonstrate actin filaments via shadow projection (*grayscale*) and the conidia as *spots* (*red*). The *right images* demonstrate the middle images arbitrarily rotated counterclockwise. **(E)** Numbers of conidia for NM, a Gr-1, and IgG2b mice. **(F–H)** Numbers of conidia in the proximal, middle, and distal regions of the conducting airways of immunocompetent **(F)**, neutropenic **(G)**, and control **(H)** mice. Data are shown as median and IQR, *n* = 8 mice per group. Significant differences between groups were determined using one-way ANOVA and Dunnett’s multiple comparison tests. *p ≤ 0.05.

To demonstrate the retention of *A. fumigatus* conidia within the bronchial branches, an analysis of whole-mount conducting airway specimens from both immunocompetent and neutropenic CD11c-EYFP mice was carried out. Specimens from control mice that received an isotype control (IgG2b) instead of depleting antibodies were also examined. To visualize the conducting airway wall—the region between the smooth muscles and the epithelial barrier—Atto-425-conjugated phalloidin was used (**Figure 4A**).

It was observed that, 48 h after o.ph. administration, *A. fumigatus* conidia were located at the luminal side of the conducting airway epithelium in immunocompetent, neutropenic, and control mice (**Figures 4B–D**). The conducting airway was divided into the proximal (to the trachea), middle, and distal regions based on the location of the next-generation airways (**Figure 4A**), as previously reported (Veres et al., 2007; Shevchenko et al., 2013). For the quantitative analysis, at least four *Z*-stacks were acquired at each region as arbitrarily indicated (**Figure 4A**).

The total number of *A. fumigatus* conidia present in the conducting airway mucosa of neutropenic mice was significantly higher compared with that of immunocompetent and control mice (**Figure 4E**). To pinpoint the precise location of the conidia, their quantities in the proximal, middle, and distal regions were estimated across immunocompetent, neutropenic, and control mice (**Figures 4F–H**). Analysis of immunocompetent and control mice revealed no significant differences in the distribution of conidia across the regions (**Figures 4F, H**). However, in the case of neutropenic mice, the number of conidia was significantly greater in the proximal region compared with the distal region (**Figure 4G**).

The increased number of *A. fumigatus* conidia detected in neutropenic mice, compared with those in immunocompetent and control animals, supports the earlier observation of conidial retention in the bronchial branches.

### 3.5 CD11c^+^ cells internalize *A. fumigatus* conidia in the main bronchus of neutropenic mice

**Figure 5:**
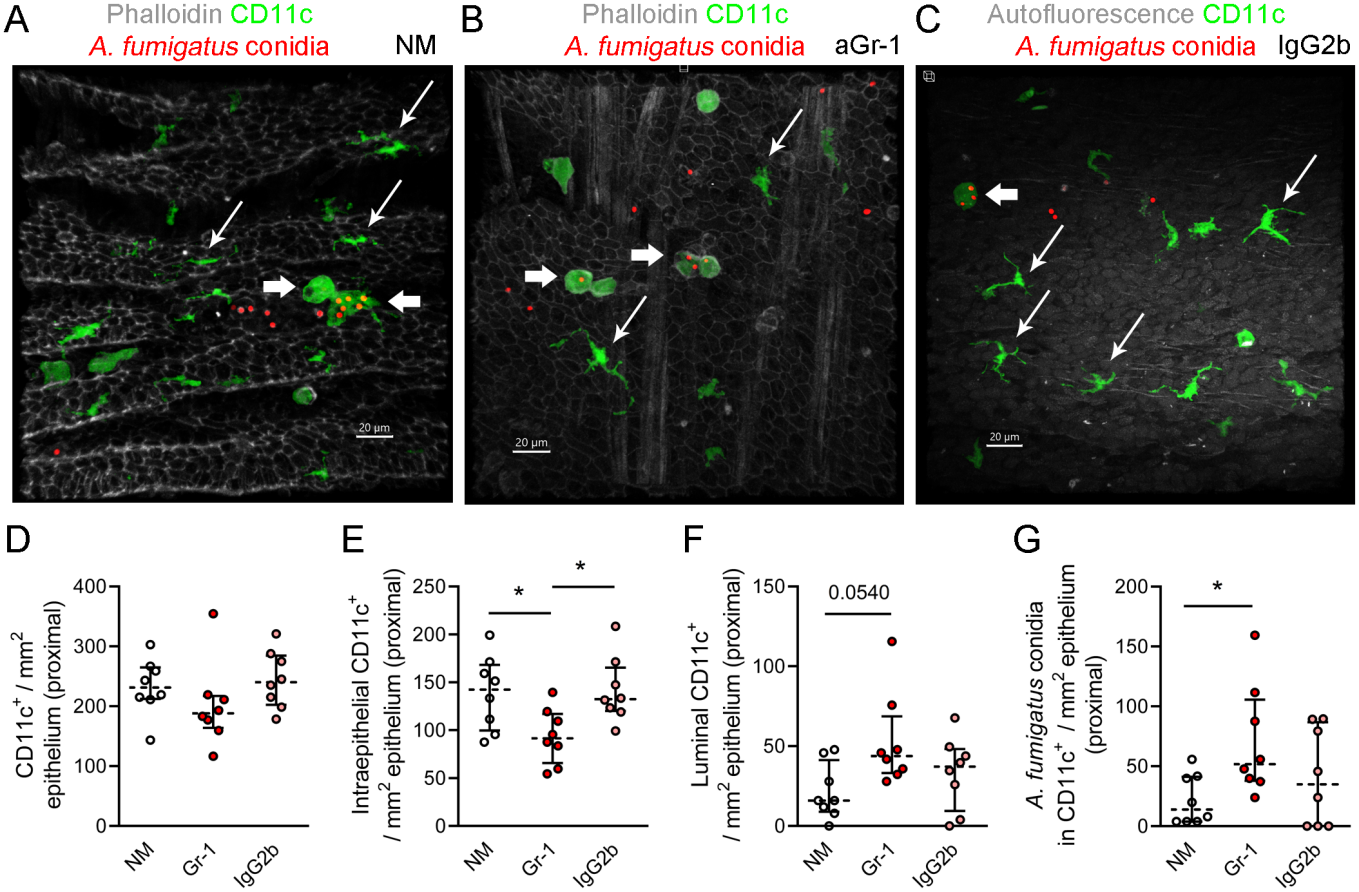
CD11c^+^ cell–*Aspergillus fumigatus* conidia interactions in the conducting airway mucosa. **(A–C)** Representative images of the region of the conducting airway mucosa of immunocompetent **(A)**, neutropenic **(B)**, and control **(C)** mice showing CD11c^+^ cells (*green*), *A*. *fumigatus* conidia (*red*), and actin fibers (*grayscale*) in the epithelium and smooth muscles **(A, B)** or tissue autofluorescence **(C)**. *Scale bar*, 20 µm. **(D–G)** Total number of CD11c^+^ cells **(D)**, intraepithelial CD11c^+^ cells **(E)**, and luminal CD11c^+^ cells **(F)** and conidia internalized by CD11c^+^ cells **(G)** in the proximal region of the conducting airway mucosa of immunocompetent (*NM*), neutropenic (*aGr-1*), and control (*IgG2b*) mice. Data are shown as median and IQR for *n* = 8 mice. Significant differences between groups were determined using one-way ANOVA and Dunnett’s multiple comparison tests. *p ≤ 0.05.

To determine whether the internalization of *A. fumigatus* conidia by phagocytic cells impacts conidial retention in the conducting airways of neutropenic mice, CD11c^+^ cell–conidia interactions were examined, focusing on the proximal region of the conducting airway. Consistent with our previous study (Bogorodskiy et al., 2020), in the conducting airway mucosa of immunocompetent mice, round-shaped CD11c^+^ cells were noted on the luminal side of the airway epithelium, as well as intraepithelial CD11c^+^ cells displaying irregular shapes and dendrites (**Figure 5A**; **Supplementary Figure S5**). These same populations were also noted in both neutropenic and control mice (**Figures 5B, C**).

Firstly, the total number of CD11c^+^ cells in the conducting airways was estimated, particularly in the proximal region of immunocompetent, neutropenic, and control mice. Significant differences were not observed (**Figure 5D**; **Supplementary Figure S6**). Interestingly, in the proximal region, the number of intraepithelial CD11c^+^ cells was significantly lower in neutropenic mice than in immunocompetent and control mice (**Figure 5E**). As previously demonstrated, 48 h after application, *A. fumigatus* conidia were found on the luminal side of the conducting airway epithelium; thus, luminal CD11c^+^ cells were of significant interest. Analysis of the proximal regions of the main bronchi revealed significant differences in the number of luminal CD11c^+^ cells between immunocompetent and neutropenic mice 48 h post-application of conidia (**Figure 5F**). At this time point, a significant difference was also observed in the number of *A. fumigatus* conidia internalized by CD11c^+^ cells in the conducting airway mucosa and specifically in the proximal region between immunocompetent and neutropenic mice (**Figure 5G**; **Supplementary Figure S7**). However, the percentages of conidia internalized by CD11c^+^ cells from the total conidia count did not significantly differ among immunocompetent, neutropenic, and control mice (**Supplementary Figure S7**). There were also no significant differences in the percentages of CD11c^+^ cells that internalized conidia among immunocompetent, neutropenic, and control mice (**Supplementary Figure S7**).

Thus, CD11c^+^ cells in the conducting airway mucosa can internalize conidia even in the absence of neutrophils. Despite the increased number of conidia found in the main bronchi of neutropenic mice compared with immunocompetent and control mice, CD11c^+^ cells are capable of internalizing an equivalent portion of conidia.

## 4 Discussion

*A. fumigatus* conidia are common in the air and, when inhaled, can penetrate the airways of both immunocompetent and neutropenic organisms. In the current study, we have advanced the visualization of *A. fumigatus* conidia by labeling them in the immunohistochemically stained, optically cleared whole mouse lung lobe of immunocompetent and neutropenic mice. This was achieved using the CLSM-based experimental setup that we have previously developed (Maslov et al., 2021). Similar to the light sheet fluorescent microscopy (Amich et al., 2020), it allows the detection of 2- to 3-μm-sized *A. fumigatus* conidia in the airways throughout the entire lung lobes. For the quantitative analysis of conidial distribution, we modified a previously developed approach (Maslov et al., 2021) that enables the accurate identification of airway generation conidial location in the main bronchus, the intermediate bronchi, and the terminal bronchioles. Although the imaging was performed with a relatively high resolution (512 × 512), the approach only allowed determining the proportion of conidia due to agglomeration rather than providing absolute numbers. Therefore, to identify absolute numbers, the precise location, and the quantitative parameters of immune cell–conidia interactions, we combined the imaging of whole lung lobes with higher-magnification imaging of the whole-mount conducting airway (Shevchenko et al., 2018; Bogorodskiy et al., 2020).

In this study, it was demonstrated that, in immunocompetent organisms, *A. fumigatus* conidia primarily settle in the alveolar space, not in the bronchial branches, without any kinetic changes within 72 h. As has been repeatedly shown, immunocompetent mice are resistant to fungal growth and can eliminate *A. fumigatus* conidia from the respiratory tract (Svirshchevskaya et al., 2009; Buskirk et al., 2014; Savers et al., 2016). We can infer from these data, as well as the fact that, in the present study, only the conidial proportions and not the total numbers were detected, that the total number of conidia in the alveolar space of immunocompetent mice would be inadequate for infection. Therefore, the smaller fraction of conidia in the bronchi may reflect the elimination of conidia from the bronchial branches by mucociliary clearance in immunocompetent mice. In contrast, in neutropenic conditions, the fraction of conidia in the bronchial branches was substantially increased compared with immunocompetent mice, indicating the impairment of mucociliary clearance. The movement of conidia from the terminal bronchioles and intermediate bronchi to the main bronchus was shown by the kinetics of conidial distribution in the bronchial generations. The findings from this study suggest that conidia are eliminated from the bronchial branches of immunocompetent, but not neutropenic, mice, as 48 h after the administration of conidia, the proportion of conidia significantly increased in neutropenic compared with immunocompetent mice.

In the current study, 5 × 10^6^ non-viable *A. fumigatus* conidia were administered o.ph. to anti-Gr1-treated mice, and approximately 60% of the conidia were detected in the bronchial branches. In another study, Amich et al. (2020) infected mice with different immunosuppressive regimens intranasally using 2 × 10^5^ of viable conidia and reported up to 80% of the conidia located in the bronchial branches. Therefore, various factors such as the dosage, conidial status, and the administration route can affect conidial distribution. Clinical reports have described tracheobronchial manifestations of invasive aspergillosis in patients subjected to extensive immunosuppressive therapy due to graft *versus* host disease or in those with hematological malignancies (Krenke and Grabczak, 2011; Janssens et al., 2024). Thus, mouse models of immunosuppression and particularly neutrophil depletion can facilitate mechanistic investigations and the development of treatment strategies for invasive tracheobronchial aspergillosis.

Physical factors influence the dissemination of airborne pathogens in the airways (Kleinstreuer et al., 2008). Mucociliary clearance aids in eliminating these pathogens from the respiratory tract (Knowles and Boucher, 2002; Roe et al., 2025). In this report, an impaired conidia clearance from the bronchi was observed in neutropenic mice. Defects in mucociliary clearance are considered an explanation for the tracheobronchial manifestations of invasive pulmonary aspergillosis in patients (Janssens et al., 2024). Mucolytics are routinely used to enhance the clearance of pathogens from conducting airways (Roe et al., 2025). Several studies have underscored the potential benefits of mucolytics for patients with aspergillosis (Henderson and Pearson, 1968; De Lucca et al., 1996; Xu et al., 2009; Otu et al., 2018). Using the approach outlined in this study, further investigation into the effects of mucolytics on the increased proportion of conidia in the bronchial branches can be conducted using the neutrophil depletion mouse model.

Another aspect of successful conidia elimination relates to immune system functions, as the immune system maintains the epithelial barrier integrity and prevents the germination of pathogens within the lung tissue (Shevchenko et al., 2013; Iwasaki et al., 2017). Upon inhalation, *A. fumigatus* conidia are situated on the luminal side of the conducting airway epithelium. In immunocompetent mice, neutrophils traverse the epithelial barrier and interact with conidia within just 6 h (Shevchenko et al., 2018). In addition, resident phagocytic cells in the luminal side of the epithelium, such as macrophages or dendritic cells (both expressing the CD11c marker), aid in the internalization of the conidia (Amich et al., 2020; Bogorodskiy et al., 2020). As we have previously demonstrated in immunocompetent mice, CD11c^+^ cells internalize not only conidia but also other corpuscular pathogens shortly after exposure (Bolkhovitina et al., 2022). This internalization may be part of a pathogen-masking strategy that prevents uncontrolled neutrophil-mediated inflammation (Uderhardt et al., 2019). In immunocompetent hosts, the contribution of CD11c^+^ cells to conidia internalization is relatively small compared with neutrophils; however, in conditions of neutropenia, these cells can participate in the compensatory antifungal immune response (Park et al., 2010; Bogorodskiy et al., 2020). Our analysis of the luminal CD11c^+^ cell numbers and their interaction with *A. fumigatus* conidia revealed an enhanced role of these cells in conidial internalization in cases of neutropenia. Upon internalization, macrophages and dendritic cells can inhibit the germination of conidia (Lother et al., 2014; Rosowski et al., 2018). Therefore, the development of therapeutic approaches focusing on removing these cells, along with the ingested conidia from the airways, warrants further investigation.

In the current study, an old but reliable model of neutrophil depletion using anti-Gr-1 antibodies was utilized. As demonstrated by us and others, a relatively low dose of anti-Gr-1 yields prolonged leukocyte, myeloid cell, and neutrophil depletion compared with rat anti-mouse Ly6G, clone 1A8. This result is achieved through a single injection, which is more convenient compared with modern, two-step depletion methods (Pollenus et al., 2019; Boivin et al., 2020; Stackowicz et al., 2020). In addition, no significant difference was found in the neutrophil counts in the conducting airway mucosa of mice that received anti-Ly6G and anti-Gr-1 24 h after the antibody injection, as well as a further 6 h after the administration of conidia (Shevchenko et al., 2018). However, we hypothesize that other leukocyte populations may be affected, suggesting that murinized anti-Ly6G antibodies could be advantageous in further studies (Olofsen et al., 2022).

Interestingly, contrary to earlier reports indicating enhanced dendritic cell (CD11c^+^CD11b^+^) recruitment to the lungs under neutropenic conditions, we observed no increase in the CD11c^+^ cell numbers in the conducting airway mucosa of neutropenic mice compared with immunocompetent ones (Park et al., 2010). This suggests the migration of these cells to other lung anatomical sites, indicating the limitation of the current study focusing on investigating the CD11c^+^ cell–*A. fumigatus* conidia interaction in the main bronchus. Another limitation concerns the absence of distinguishing populations of CD11c^+^ phagocytic cells interacting with *A. fumigatus* conidia in the conducting airway mucosa. Additional staining against MHC II, CD169, and CD170 (Siglec F) could be performed to discriminate between macrophages and dendritic cells in further investigations (Svedova et al., 2017; Amich et al., 2020; Bošnjak et al., 2022; Tang et al., 2022). Staining directed to such markers as XCR1 and CD172 should be adopted in immunohistochemistry to identify certain dendritic cell populations in the conducting airway mucosa (Guilliams et al., 2016).

In the current study, the prevalence of *A. fumigatus* conidia sedimentation was observed in the bronchial branches when neutropenia is present. It was also discovered that, in the absence of neutrophils, luminal CD11c^+^ phagocytic cells facilitate the ingestion of conidia retained in the conducting airway mucosa. Collectively, these findings underscore the crucial role of resident immune cells in the conducting airway mucosa in preventing the dissemination of *A. fumigatus* conidia in conditions of neutropenia.

## Data Availability

The original contributions presented in the study are included in the article/**Supplementary Material**. Further inquiries can be directed to the corresponding author.
