# Supplementary material for: Impact of CD11c+ cells in conducting airway lumen on Aspergillus fumigatus conidia deposition in neutropenic mice
Source: Front Fungal Biol. 2025 Jun 18;6:1591891. doi: 10.3389/ffunb.2025.1591891 (PMC12213632; doi:10.3389/ffunb.2025.1591891)
Supplement: Supplementary file 1 [file DataSheet1.pdf]

Supplementary Materials

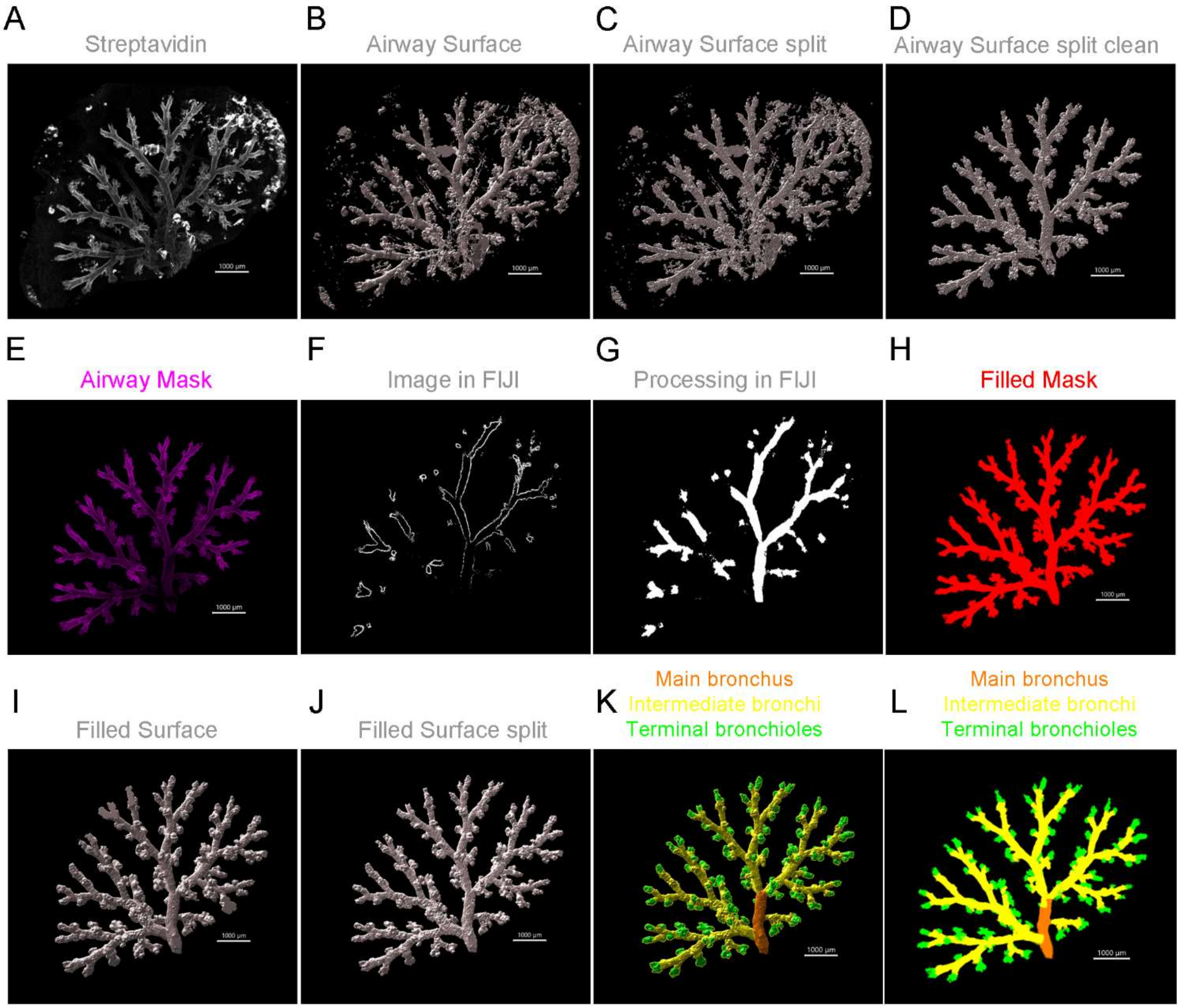

Supplementary Figure 1. Image processing sequence for the airway generation classification. **A**. Representative image of the mouse lung lobe stained with Alexa Fluor 488-conjugated streptavidin (grayscale) and underwent optical clearance. **B**. The surface of the airways was created based on the maximum intensity projection in the Alexa Fluor 488 channel. **C**. The surface of the airways was split into elements of an approximate diameter of 20 µm **D**. The excessive elements (vessels, pleura, and nonspecific tissue autofluorescence) were removed. **E**. The airway mask was created based on a clean surface. **F**. Surface was transferred to FIJI and presented as a binary image. **G**. The image was processed in FIJI using functions “Dilate”, “Fill holes”, and “Erode”. **H**. Filled mask was transferred from FIJI to Imaris. **I**. The surface was created based on the filled mask. **J**. The filled surface was split into elements of 20 µm. **K**. Elements were classified into the main bronchus, intermediate

bronchi, and terminal bronchioles. **L**. Masks of the surfaces of the main bronchus, intermediate bronchi, and terminal bronchioles were created. Scale bar 1000 μm.

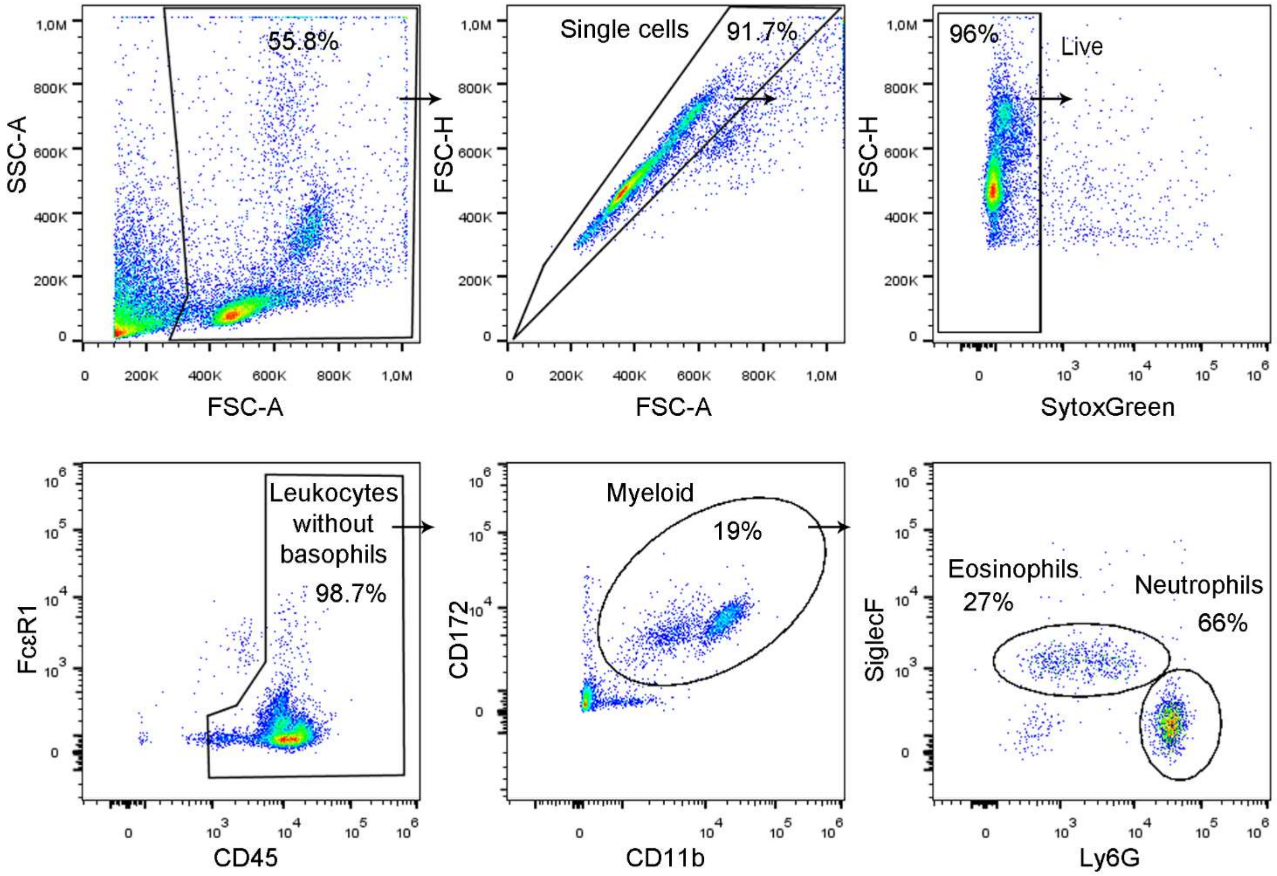

Supplementary Figure 2. Gating strategy for identifying blood myeloid cell and neutrophil populations. Representative flow cytometry plots showing gating of peripheral blood cells according to morphology, single, live, leukocytes, myeloid cells, and neutrophils.

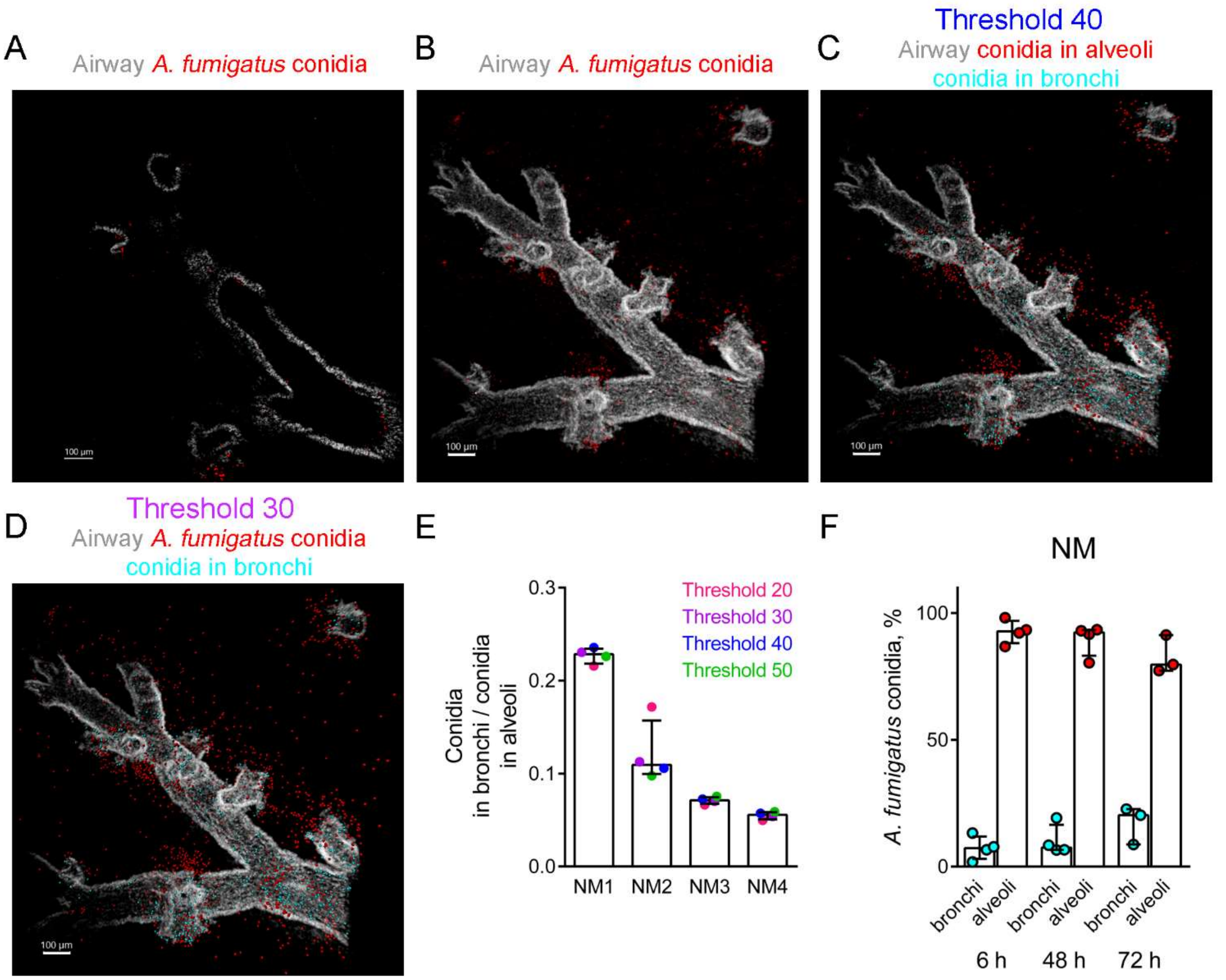

Supplementary Figure 3. Distribution of *Aspergillus fumigatus* conidia between bronchial branches and alveoli. **A**. Representative single image from a Z-stack presented as a maximum intensity projection of the airway (grayscale) and *A. fumigatus* conidia (red). **B**. Representative image of a Z-stack presented as a 3D view demonstrating airway (grayscale) and *A. fumigatus* conidia (red) via volume rendering. **C**, **D**. The image shown in (**B**) demonstrates conidia in the bronchi (cyan) and alveoli (red) as spots depicted with threshold 40 (**C**) and 30 (**D**). Spots are presented with a radius scale of 2 µm. Scale bar 100 µm. **E**. Ratios of conidia in bronchi to conidia in alveoli calculated with different thresholds: 20 (magenta), 30 (violet), 40 (blue), and 50 (green) for four immunocompetent mice (NM1, NM2, NM3, and NM4). Notably, for NM2 threshold 20 was out of the plateau region. **F**. Percentages of *A. fumigatus* conidia in the bronchial branches (cyan) and alveoli (red) from the total conidia numbers are presented for immunocompetent mice at 6, 48, and 72 h after the application. Data are shown as median and IQR for at least 3 mice per group.

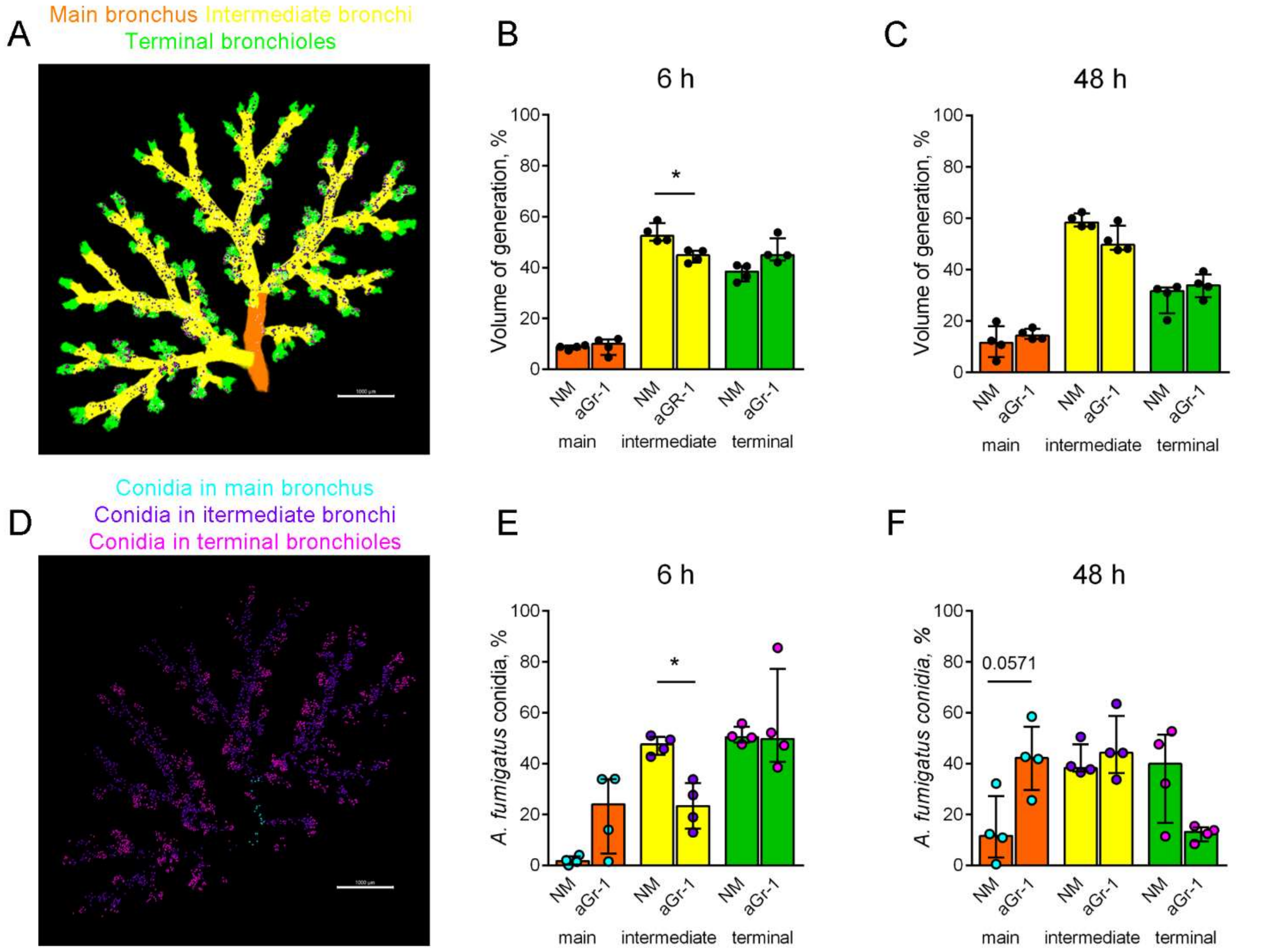

Supplementary Figure 4. Distribution of *Aspergillus fumigatus* conidia in the airway generations. **A**. Representative image of masks of airway generations: the main bronchus (orange), intermediate bronchi (yellow), and terminal bronchioles (green) and *A. fumigatus* conidia (spots) colocalized with the generations. Scale bar 1000 μm. **B**, **C**. Percentages of the volume of the main bronchus (orange bars), intermediate bronchi (yellow bars), and terminal bronchioles (green bars) from the total volume of the airway of immunocompetent (NM) and neutropenic (aGr-1) mice 6 (B) and 48 h (**C**) after conidia administration. **D**. Representative image of *A. fumigatus* conidia demonstrated as spots colocalized with the main bronchus (cyan), intermediate bronchi (violet), and terminal bronchioles (magenta). Scale bar 1000 μm. **E**, **F**. Percentages of conidia colocalized with main bronchus (orange bars), intermediate bronchi (yellow bars), and terminal bronchioles (green bars) from total conidia number in bronchi of immunocompetent (NM) and neutropenic mice (aGr-1) 6 (**E**) and 48 h (**F**) after conidia administration. Data are shown as median and IQR for n = 8 mice per group; pair-wise comparison of the indicated groups was performed using the Mann-Whitney test *: $p \leq 0.05$.

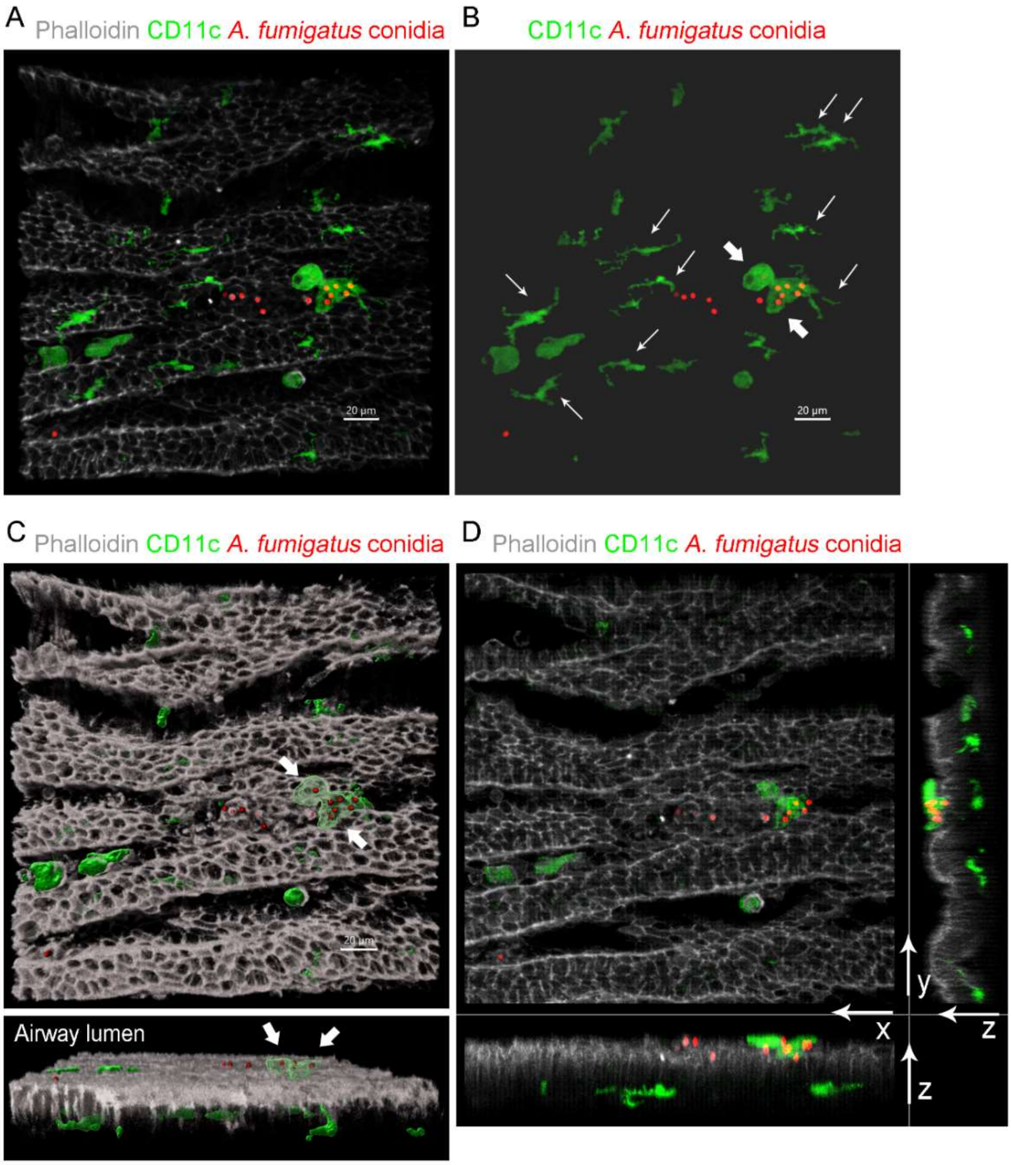

Supplementary Figure 5. Luminal and intraepithelial CD11c^{+} cells in conducting airway mucosa of mice. **A**. Representative image of the region of conducting airway mucosa of an immunocompetent mouse 48 h after *A. fumigatus* conidia application. Conidia (red), CD11c^{+} cells (green), and actin (grayscale) are shown via volume rendering. Scale bar 20 µm. **B**. The image demonstrated in (**A**) is without an actin channel. Intraepithelial CD11c^{+} cells are indicated with thin arrows, and luminal CD11c^{+} cells that internalize conidia are indicated with bold arrows. **C**. The image presented in (**A**) shows CD11c^{+} cells and epithelium via surface rendering and conidia as spots. The lower image shows a side projection. Luminal CD11c^{+} cells that internalize conidia are indicated with bold arrows. **D**. The image demonstrated in (**A**) is presented as frontal (X-Y) and side (X-Z, and Y-Z) projections.

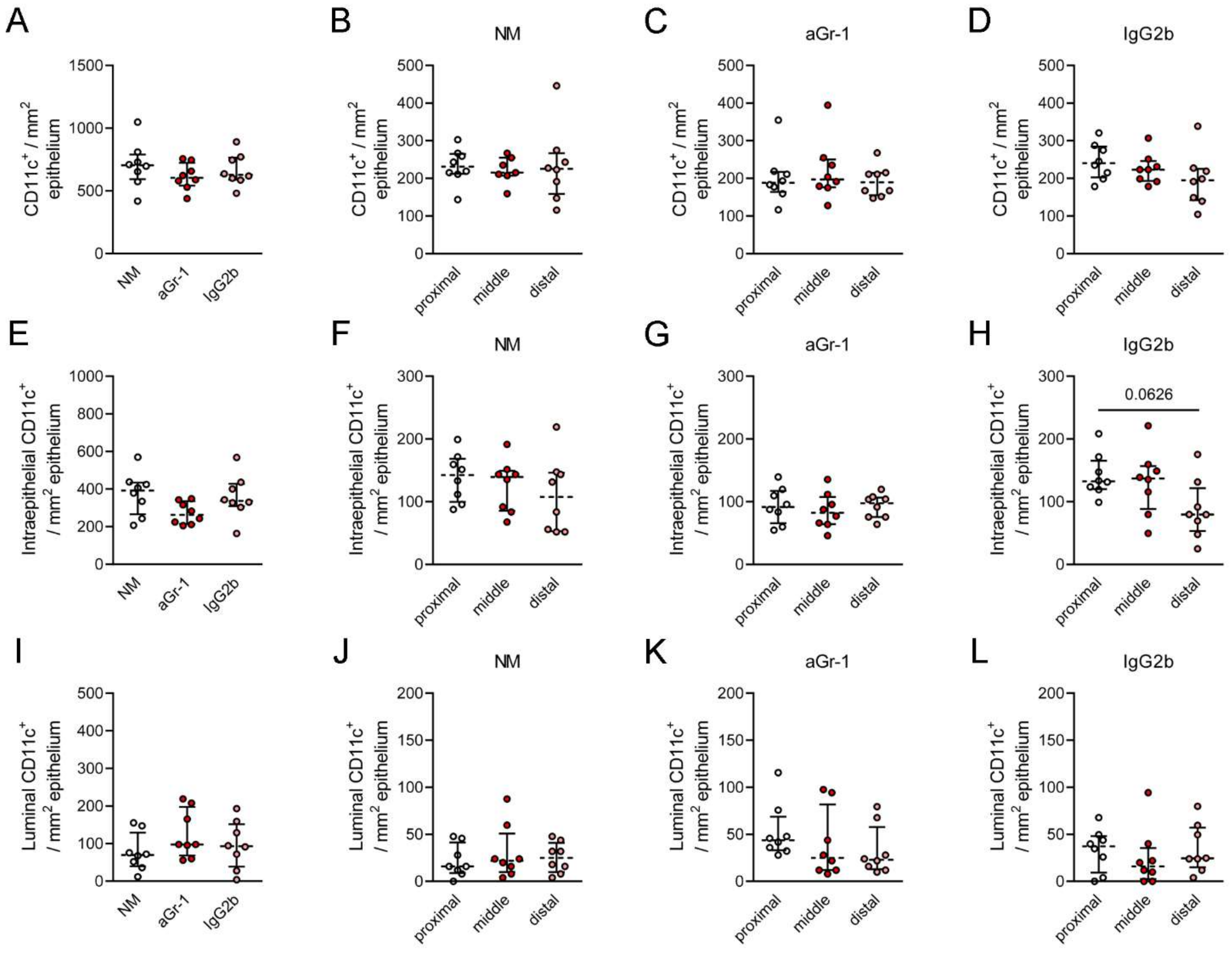

Supplementary Figure 6. Distribution of CD11c$^+$ cells in conducting airway mucosa of immunocompetent (NM), neutropenic (aGr-1), and control (IgG2b) mice 48 h after the administration of *Aspergillus fumigatus* conidia. **A**. Total numbers of CD11c$^+$ cells in the conducting airway mucosa. **B-D** The numbers of CD11c$^+$ cells in the proximal, middle, and distal regions of the main bronchus. **E**. The numbers of intraepithelial CD11c$^+$ cells. **F**-**H**. The numbers of intraepithelial CD11c$^+$ cells in the proximal, middle, and distal regions of the main bronchus. **I**. The number of luminal CD11c$^+$ cells in the conducting airway mucosa. **J-L**. The numbers of luminal CD11c$^+$ cells in the proximal, middle, and distal regions of the main bronchus. The data are shown as median and IQR for n=8 mice per group.

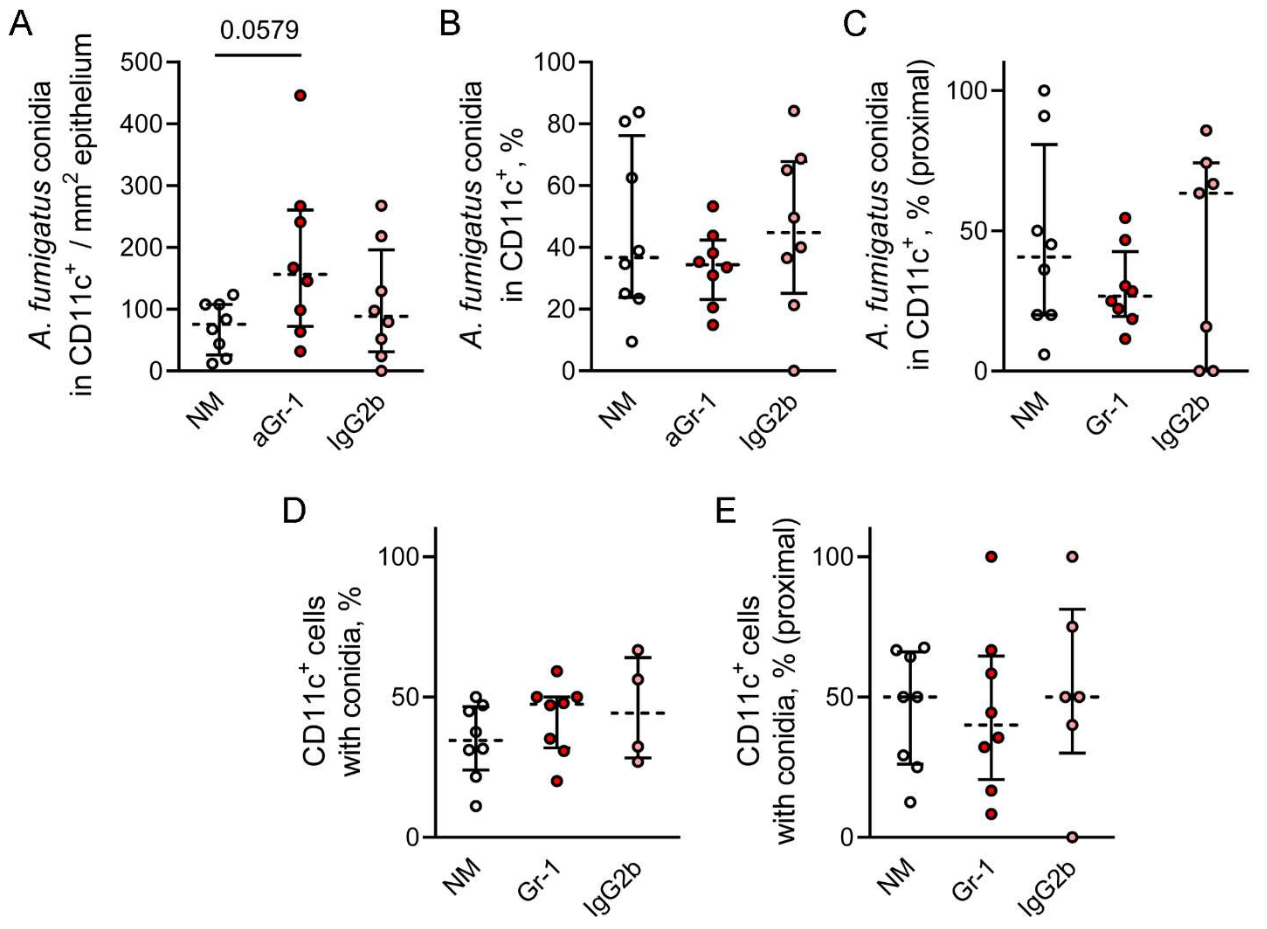

Supplementary Figure 7. Internalization of *Aspergillus fumigatus* conidia by CD11c$^+$ cells in conducting airway mucosa of immunocompetent (NM), neutropenic (aGr-1), and control (IgG2b) mice 48 h after conidia administration. **A**. The numbers of *A. fumigatus* conidia in CD11c$^+$ cells in conducting airway mucosa. **B, C**. Percentages of *A. fumigatus* conidia internalized by CD11c$^+$ cells from the total conidia numbers in conducting airway mucosa (**B**) or in the proximal region of the main bronchus (**C**). **D**, **E**. Percentages of CD11c$^+$ cells that internalized conidia from the total numbers of CD11c$^+$ cells in conducting airway mucosa (**D**) and in the proximal region of the main bronchus (**E**). The data are shown as median and IQR for n=8 mice per group.
